# Synthesis, Molecular Docking Studies, In Vitro Antimicrobial and Antifungal Activities of Novel Dipeptide Derivatives Based on *N*-(2-(2-Hydrazinyl-2-oxoethylamino)-2-oxoethyl)-Nicotinamide

**DOI:** 10.3390/molecules23040761

**Published:** 2018-03-27

**Authors:** Gaber Moustafa, Hemat Khalaf, Ahmed Naglah, Asma Al-Wasidi, Nawal Al-Jafshar, Hassan Awad

**Affiliations:** 1 Peptide Chemistry Department, Chemical Industries Research Division, National Research Centre, Dokki 12622, Cairo, Egypt; amnaglah@gmail.com; 2 Chemistry Department, College of Science and Arts-Qurayat, Jouf University, Saudi Arabia; hematsalama2016@gmail.com; 3 Photochemistry Department, Chemical Industries Research Division, National Research Centre, Dokki 12622, Cairo, Egypt; 4 Department of Pharmaceutical Chemistry, Drug Exploration and Development Chair (DEDC), College of Pharmacy, King Saud University, Riyadh 11451, Saudi Arabia; 5 Department of Chemistry, College of Science, Princess Nourah Bint Abdulrahman University, Riyadh 11671, Saudi Arabia; asmachem7@hotmail.com (A.A.-W.); Nawal.Aljafshar@hotmail.com (M.-A.-J.); 6 Chemistry of Natural and Microbial Products Department, Pharmaceutical and Drug Industries Div., National Research Centre, Dokki 12622, Cairo, Egypt; awadmhassan@yahoo.com

**Keywords:** nicotinoyl chloride, amino acids, linear dipeptides, antimicrobial activity, molecular docking studies

## Abstract

A series of linear dipeptide derivatives (**4**–**10**) were prepared and evaluated as antimicrobial agents via the synthesis of *N*-(2-(2-hydrazinyl-2-oxoethylamino)-2-oxoethyl) nicotinamide (**4**). Compound **4** was reacted with 4-chlorobenzaldehyde or 4-hydroxybenzaldehyde, to give the hydrazones **5** and **6**, respectively. On the other hand, Compound **4** was coupled with phenylisocyanate or methylisothiocyanate to give Compounds **7** and **8**, respectively. The latter compounds (**7** and **8**) were coupled with chloroacetic acid to give oxazolidine (**9**) and thiazolidine (**10**), respectively. The newly synthesized dipeptide compounds were confirmed by means of their spectral data. The antimicrobial activity of the newly synthesized compounds **4**–**10** was evaluated by agar well diffusion, and they showed good activity. Compounds **4**, **5**, and **9** gave the most promising activity in this study. Most of the tested compounds possessed MIC values ranging from 50 to 500 µg/mL. Furthermore, docking studies were carried out on enoyl reductase from *E. coli* and cytochrome P450 14 α-sterol demethylase (Cyp51) from *Candida albicans* active sites. The MolDock scores of the seven tested compounds ranged between −117 and −171 and between −107 and −179, respectively.

## 1. Introduction

Nicotinic acid (also known as pyridine-3-carboxylic acid, vitamin B3, and niacin) is a widely used in the food, biochemical, and pharmaceutical industries. It is found in various plants and animals [1] and plays a vital role in biological processes such as the production of energy [2]. Nicotinic acid supplements (astatin drug) have not been found useful for decreasing the risk of cardiovascular disease [3] but appear to be highly effective in those not taking a statin, such as ezetimibe, simvastatin, gemfibrozil, cholestyramine, colestipol, or clofibrate [4]. Although nicotinic acid and nicotinamide are similar in their vitamin activity, nicotinamide does not have the same pharmacological properties and lipid-modifying effects as nicotinic acid. However, some of the new heterocyclic and peptide derivatives have been studied with respect to antiviral [5], anti-inflammatory [6], enzymatic peptide [7], and antimicrobial activities [8,9]. Additionally, there have been several successful publications of peptide synthesis functioning as an antimicrobial for therapeutic applications [10]. A large number of antimicrobial peptides show highly broad-spectrum activities against different microorganisms, Gram-positive and Gram-negative bacteria, fungi, and viruses [11]. Many peptide candidates have antimicrobial properties against multiple drug resistances and possess a low tendency for the improvement of said resistances [12,13,14]. Peptides are being used as therapeutic agents such as Lupron™, Sandostatin™, and Zoladex™ [15,16]. Peptides are intrinsically able to interact with biological systems and are therefore potent therapeutics [17,18,19]. Due to the importance of nicotinic acid derivatives, amino acids and peptides act as bioactive compound. The objective of this study was to prepare novel antimicrobial dipeptide compounds that are based on *N*-(2-(2-hydrazinyl-2-oxoethylamino)-2-oxoethyl)-nicotinamide.

## 2. Results and Discussion 

### 2.1. Chemistry

**Scheme 1:**
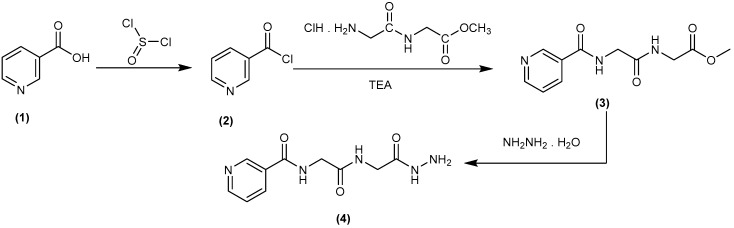
Synthetic routes for *N*-(2-(2-hydrazinyl-2-oxoethylamino)-2-oxoethyl)-nicotinamide (**4**).

In our previous studies of peptide candidates, good antimicrobial properties [20,21,22,23,24] and anticancer activities [25,26,27,28], as well as, pyrazolo derivatives as anticancer agents [29]. Thus, this study aims to conjugate dipeptides and nicotinic acid. Some of the novel derivatives (**5**–**10**) were synthesized based on nicotinyl-glycyl-glycine-hydrazide (**4**), which may be expected to possess various antibacterial and antifungal properties. Accordingly, a rational design, synthesis, purification, and structural characterization of nicotinyl-glycyl-glycine-hydrazide, were optimally synthesized via conventional synthetic peptide coupling methods (in solution). The compound nicotinyl-glycyl-glycine-hydrazide (**4**) was synthesized by the conversion of nicotinic acid (**1**) to nicotinoyl chloride (**2**) through thionyl chloride, while Compound **2** was then coupled with a free glycyl-glycine methyl ester to give glycyl-glycine-methylester (**3**). IR of Compound **3** showed absorption bands at 3400 cm^−1^ due to NH in addition to absorptions of carbonyl groups at 1734, 1660, and 1580 cm^−1^, respectively. ^1^H-NMR of the ester (**3**) revealed two signals in the region δ 3.7–3.3 of 2CH_2_ in addition to the D_2_O exchangeable signals of amidic. Hydrazinolysis of Compound **3** with hydrazine hydrate (99%) led to the corresponding dipeptide hydrazide (**4**) in 65% yield (Scheme 1). ^1^H-NMR of hydrazide (**4**) exhibited D_2_O exchangeable signals at δ 10.67, 9.04, 8.99, and 8.50 in addition to the signals of 2CH_2_ at δ 3.8–3.5. The mass spectrum of Compound **4** showed a peak equal to its molecular weight at *m*/*z* = 252 (M^+^ + 1).

**Scheme 2:**
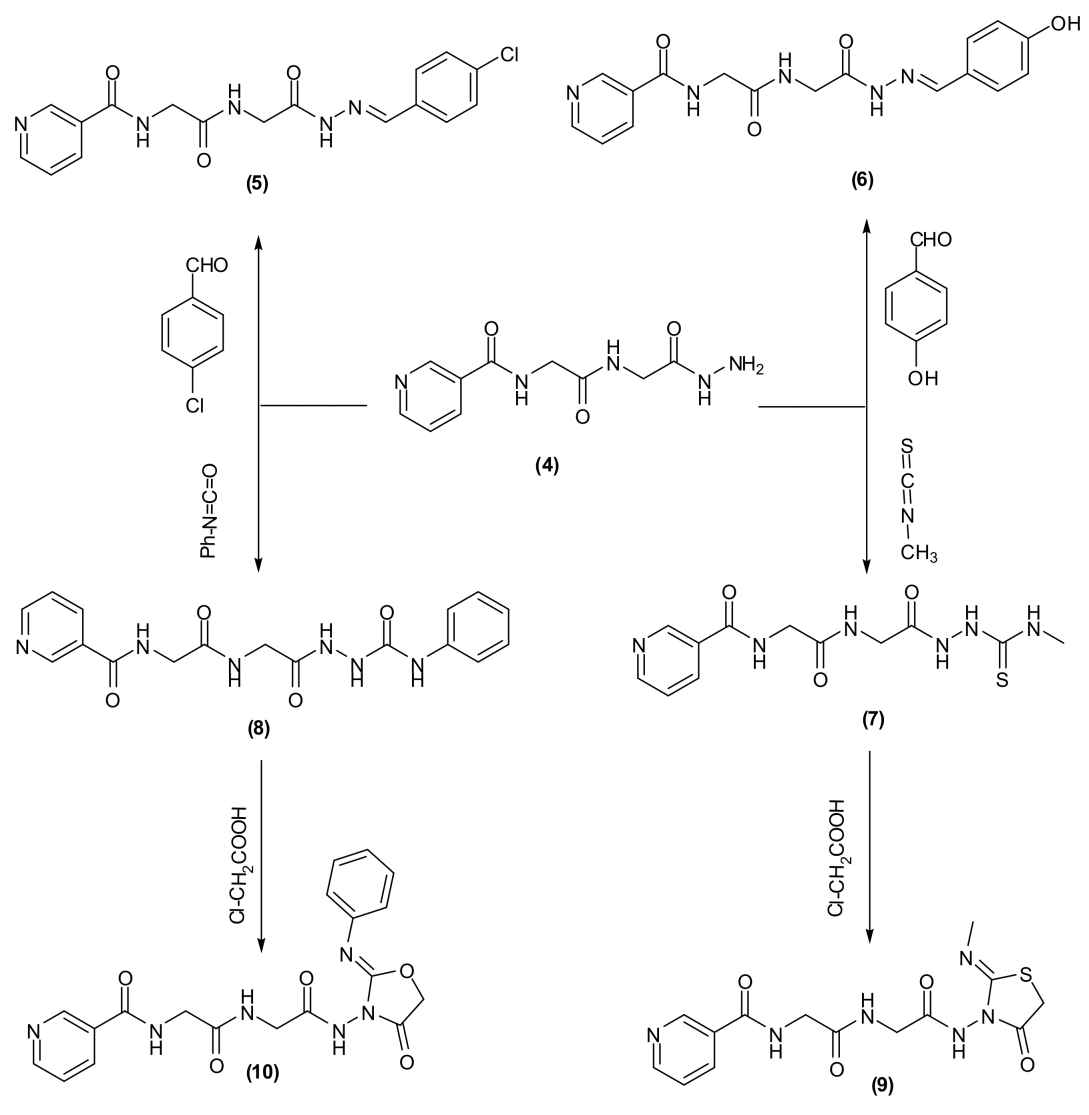
Synthetic routes for *N*-(2-(2-hydrazinyl-2-oxoethylamino)-2-oxoethyl)-nicotinamide derivatives (**5**–**10**).

The condensation of the *N*-(2-(2-hydrazinyl-2-oxoethylamino)-2-oxoethyl)-nicotinamide, (Compound **4**) with 4-chlorobenzaldehyde or 4-hydroxybenzaldehyde took place through refluxing in ethanol using piperidine as an organic base. This produced substituted hydrazides (**5**,**6**) (Scheme 2). ^1^H-NMR of the hydrazones **5** and **6** showed the characteristic signal of –N=CH– in the range δ 8.36–8.16.

On the other hand, Compound **4** was reacted with methyl isothiocyanate or phenylisocyanate in dry dioxane, which produced hydrazinecarbothioamide or hydrazinecarboxamide derivatives (**7**,**8**), respectively (Scheme 2). The structure of Compounds **7** and **8** was established under the basis of their spectral data. Compounds **7** and **8** were reacted with chloroacetic acid in ethanol to give thiazolidine or oxazolidin derivatives (**9**,**10**), respectively (Scheme 2). ^1^H-NMR spectra showed characteristic singlets at δ 4.45 (CH_2_ of thiazolidine ring), 3.17, 2.95 (2CH_2_ of α-gly) for Compound **9** and at δ 4.29 (CH_2_ of oxazolidine ring), 3.67, 3.05 (2CH_2_ of α-gly) for Compound **10**.

### 2.2. Biological Evaluations

The antibacterial and antifungal activities of the synthesized Compounds **5**–**10** and the starting Compound **4** against a panel of pathogenic tested organisms are represented in Table 1. The results reveal that some of these synthesized candidates are highly biologically active with different spectrum activities.

The results in Table 1 showed variations in antimicrobial effect against each test pathogenic microorganisms. In the case of *Bacillusubtilis*, Compounds **4** and **9** showed a strong inhibitory effect, and an inhibition zone (IZ) diameters of 29 mm and 30 mm were recorded. The other compounds recorded a moderate inhibitory effect and ranged from 16–20 mm in comparison to the antibiotic drug used as the standard antibacterial in this study. On the other hand, in the case of *E. coli*, Compound **4** showed a strong inhibitory effect, and Compounds **6** and **9** showed a weak inhibitory effect as well. The other tested compounds recorded a moderate inhibition effect and ranged from 17–20 mm in comparison to the reference drug. 

By testing the compounds against *Candida albicans*, Compounds **4**, **5,** and **9** showed strong inhibitory effects and had IZ diameters of 25–30 mm. Moreover, Compounds **6**, **7**, **8**, and **10** showed a moderate inhibition effect, and the IZ diameters ranged between 16 and 18 mm in comparison to the antifungal drug that was used in this study. In the case of the pathogenic fungi *Aspergillus niger*, most of the compounds had a negative effect against the test pathogen except Compounds **4** and **5**, which showed a moderate inhibition effect and ranged from 16–20 mm.

Finally, all compounds of this series, the newly synthesized Compounds **4**–**10**, displayed activity against Gram-positive, Gram-negative, and fungi. The results for the inhibitory activity represented a promising indication in Gram-positive, Gram-negative, and fungi pathogens.

**Figure 1:**
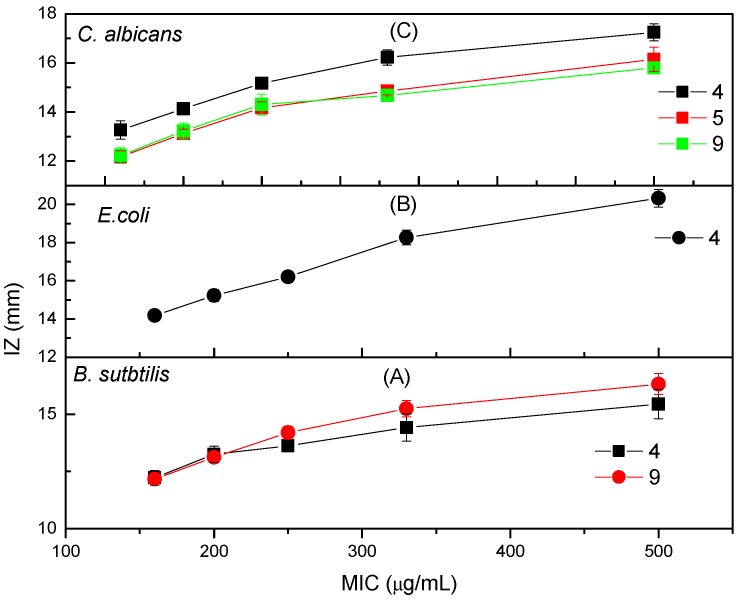
The minimum inhibitory concentrations (MICs) of Compounds **4**, **5**, and **9** against differently tested microorganisms expressed as inhibition zones of growth in millimeters.

The minimum inhibitory concentration (MIC) of the newly synthesized peptide derivatives are presented in (Table 1 and Figure 1a–c). The MIC was 160 µg/mL based on the compounds tested, but the inhibition zones obtained were different and ranged from 12–14 mm. The MIC of Compound **4** was 160 µg/mL against *B. subtilis*, *E. coli*, and *C. albicans*. The MIC of *N*-(2-(2-hydrazinyl-2-oxoethylamino)-2-oxoethyl)-nicotinamide (**5**) was 160 µg/mL against *B. subtilis*, and the inhibition zone was 12 mm. Finally, the MIC of *N*-(2-oxo-2-(2-oxo-2-(2-(phenylcarbamoyl)-hydrazinyl)-ethylamino)-ethyl)-nicotinamide (**9**) was 160 µg/mL against *B. subtilis* and *C. albicans*, and the inhibition zone obtained was 12 mm.

### 2.3. Molecular Docking Studies

Molecular docking studies are currently gaining much attention in the field of medicinal chemistry. The significant experimental antibacterial and antifungal activities of Compounds **4**–**10** provide a hint to performing molecular docking studies to understand the protein–ligand interactions. The docking was performed via the Molegro Virtual Docker for the active sites of enoyl reductase from *E. coli* (PDB 1C14) and cytochrome P450 14-α-sterol demethylase (Cyp51) from *Candida albicans* (PDB 5TZ1) in order to predict the binding mode and support the biological results. Bacterial enoyl reductase catalyzes an important step in bacterial fatty acid biosynthesis. Enoyl reductase is an attractive target for antibacterial drug discovery because of its essential role in metabolism and its sequence conservation across many bacterial species. On the other hand, cytochrome P450 14α-sterol demethylase (Cyp51) catalyzes the oxidative removal of the 14-α-methyl group of lanosterol and/or eburicol in fungi by a typical P450 mono-oxygenase activity. This protein contains an iron protoporphyrin moiety located at the active site, and our compounds may bind to the iron atom via a nitrogen atom in the nicotinamide ring. After reviewing the different chemical classes that docked into the enoyl reductase active site, such as amides, triazoles, azoles, and pyridone derivatives [30,31,32,33], we examined our compounds on the active site because some structural similarities exist. The same was done with respect to the cytochrome P450 14-α-sterol demethylase active site, which was examined with different groups of compounds from different chemical classes, such as imidazoles, pyrimidines, triazoles, thiosemicarbazides, and acetamide derivatives [34,35,36,37].

**Figure 2:**
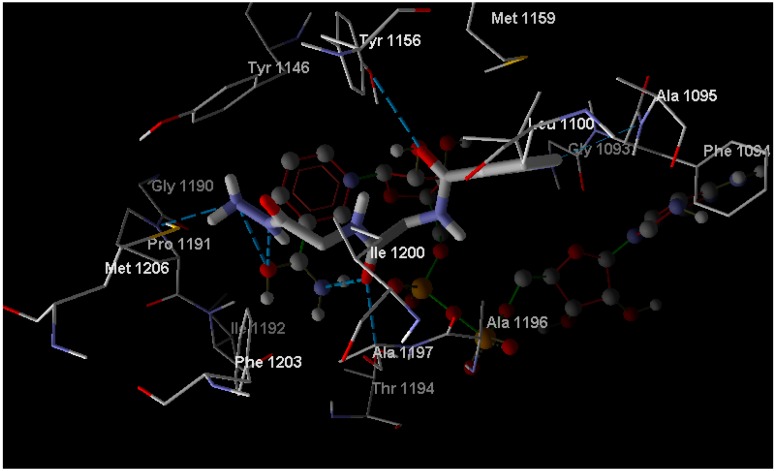
A binding mode of **4** into the binding site of enoyl reductase (Red: Oxygen, Blue: Nitrogen, Gray: Carbon, Dash line: Hydroegen bonds).

**Figure 3:**
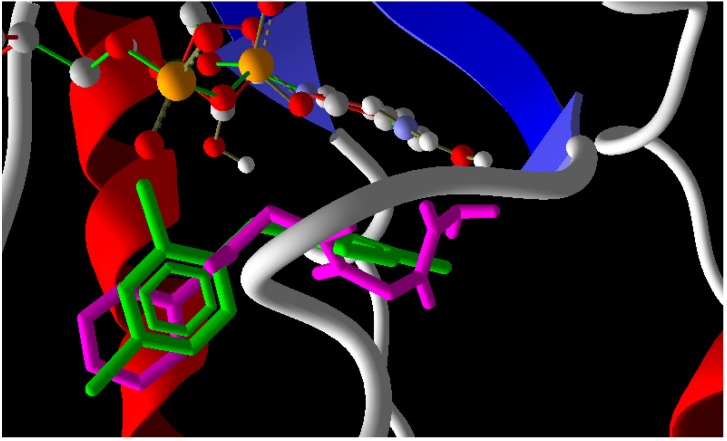
Overlay of Compound **4** (purple) and triclosan (green) in the groove of enoyl reductase (Red: alpha helix Blue: Beta sheets).

**Figure 4:**
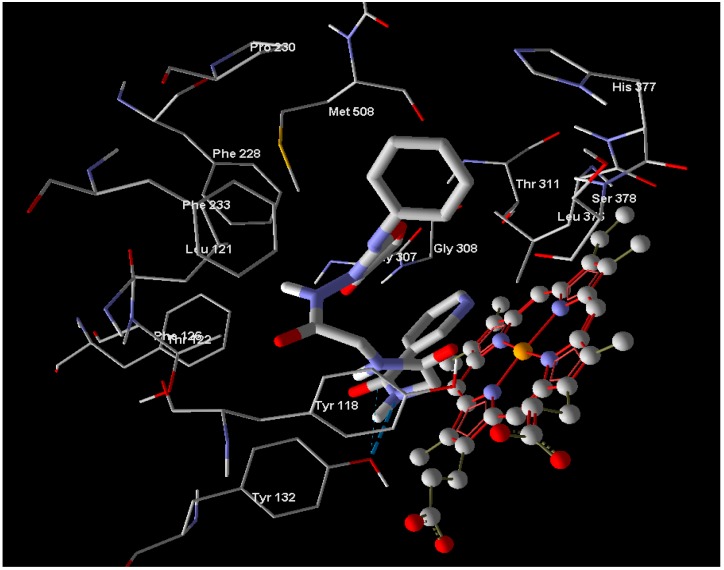
Binding mode of Compound **10** into the binding site cytochrome P450 14-α-sterol demethylase (Red: Oxygen, Blue: Nitrogen, Gray: Carbon, Dash line: Hydroegen bonds).

**Figure 5:**
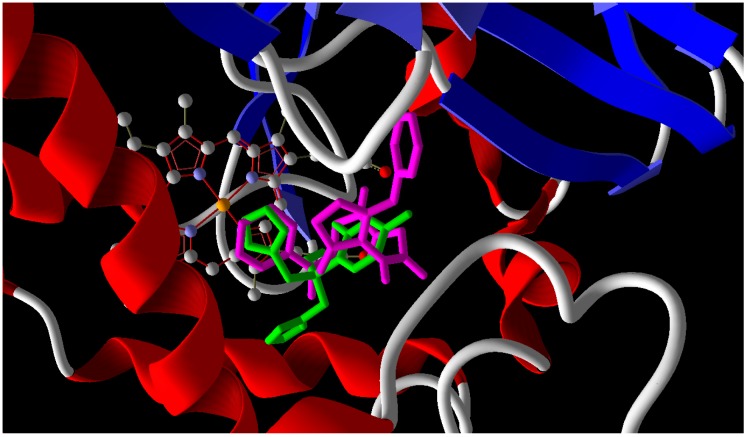
Overlay of Compound **10** (purple) and fluconazole (green) in the groove of cytochrome P450 14-α-sterol demethylase (Red: alpha helix Blue: Beta sheet).

In the case of enoyl reductase from *E. coli*, the seven compounds interact with the amino acids of its active site and give MolDock scores ranging from −171 to −117 (Table 2). The most stable complex was formed with Compound **10**, but Compound **4**, which has the best biological activity, makes four hydrogen bonds with the Ala1095, Tyr1156, Pro1191, and Thr1194 amino acids and four hydrogen bonds with the NAD+ coenzyme (Figure 2). Compound **4** and the reference drug (triclosan) were superimposed in the active site as shown in Figure 3. On the other hand, the docked compounds on the active site of cytochrome P450 14-α-sterol demethylase also gave MolDock scores ranging from −179 to −107 (Table 2), which is slightly better than those of the enoyl reductase active site. Compound **10** also has the best Moldock score (−179) and forms two hydrogen bonds with Tyr132 amino acid and hydrophobic interactions with Thr118, Phe126, Phe228, and Phe233 (Figure 4). Compound **10** and fluconazole (the reference drug) were superimposed in the active site as shown in Figure 5. This superior interaction between Compound **10** and the enzymes active sites can be explained by the increase in the chemical building of the molecule, which makes extra bonding with amino acid residues of the active site.

## 3. Materials and Methods 

### 3.1. Chemistry 

The solvents, chemicals, and thin layer chromatography used in this work were obtained from international chemical companies: Sigma (Ronkonkoma, NY, USA), Fluka (Buchs, Switzerland), and E. Merck (Hohenbrunn, Germany). The melting points were determined using the Digital Electro thermal melting point apparatus in opened glass capillary tubes and are uncorrected. Elemental micro-analyses for carbon, nitrogen, and hydrogen (Micro-Analytical Unit, Cairo University, Cairo, Egypt) were obtained within good limits of the theoretical values (±0.4%). Infrared (IR) spectra were listed as KBr disks using the Fourier transform infrared spectrophotometer (Shimadzu; Model: IRAffinity-1S) at the Micro-Analytical Unit at Cairo University in Egypt. The measurements of mass spectra occurred on a gas chromatograph–mass spectrometer (Shimadzu, Kyoto, Japan; Model: QP2010 ultra) at the Micro-analytical Unit at Cairo University in Egypt. The ^1^H-NMR and ^13^C-NMR spectra were run on JEOL, JöEL500 MHz instruments (Tokyo, Japan) in DMSO-*d*_6_.

*Synthesis of nicotinoyl chloride* (**2**) *and glycyl-glycine-methyl-ester-hydrochloride*: These compounds were prepared according to previously reported methods [38,39]. 

*Synthesis of nicotinyl-glycyl-glycine-methyl ester* (**3**): A cold dichloromethane (DCM) solution of free glycyl-glycine methyl ester (4.13 gm, ~30 mmol, −20 °C) was added carefully under control conditions to a cold DCM solution of the nicotinoyl chloride (**2**) (−20 °C, 3 gm, 14.78 mmol). The reacted mixture was stirred for 3 h at −20 °C and subsequently for 24 h at room temperature. It was then washed with water, 1 N sodium bicarbonate, and 1 N potassium hydrogen sulfate followed by water again and then dried over sodium sulfate. Next, the solvent was evaporated and the obtained compound was solidified by petroleum ether (boiling point: 40–60 °C). The solid was filtered off, dissolved in methanol (MeOH), and precipitated by petroleum ether to give Compound **3**.

*Methyl-2-(2-(nicotinamido)-acetamido)-acetate* (**3**): Yield: 80%; melting point: 122–124 °C, IR (cm^−1^): (KBr): ν = 3400 (NH stretching), 3030 (CH, aromatic), 2900 (CH, aliphatic), 1734 (C=O, ester), 1660, and 1580 (C=O amide I and II, respectively). ^1^H-NMR (500 MHz, δ, ppm, DMSO-*d*_6_): δ = 9.22−9.00 (s, 1H, 1NH, D_2_O exchangeable, amide), 8.90 (1H, aromatic H, C_1_, Pyr.), 8.81 (1H, aromatic H, C_5_, Pyr.), 8.65 (1H, aromatic H, C_3_, Pyr.), 8.30 (s, 1H, 1NH, D_2_O exchangeable, Py–CONH amide), 7.30 (1H, aromatic H, C_4_, Pyr.), 3.7–3.3 (m, 7H, 2CH_2_ α-gly + MeO). MS (EI, 70 eV): *m*/*z* (%) = 251 (M^+^, 3.55), 220 (1.66), 160 (11.44), 78 (12.90), 63 (100), 50 (2.00).Molecular formula (molecular weight): C_11_H_13_N_3_O_4_ (251.2). Calculated analysis: C, 52.59; H, 5.22; N, 16.73; Found: C, 52.62; H, 5.25; N, 16.70.

*Synthesis of nicotinyl-glycyl-glycine-hydrazide* (**4**): To a stirred methanolic solution (50 mL) of the corresponding dipeptide methyl ester (**3**) (1 mmol), hydrazine hydrate 99% (0.35 mL, 10 mmol) was added. The mixture was then refluxed for 3 h, the solvent was evaporated and the obtained residue was triturated with diethylether, filtered off, and precipitated from MeOH/diethylether to give nicotinyl-glycyl-glycine-hydrazide (**4**).

*N-(2-(2-Hydrazinyl-2-oxoethylamino)-2-oxoethyl)-nicotinamide* (**4**): Yield: 65%; melting point: 261–263 °C, IR (cm^−1^): (KBr): ν = 3425 (NH stretching), 2997 (CH, aromatic), 2913 (CH, aliphatic), 1655, 1542 and 1534 (C=O amide I, II, and III, respectively). ^1^H-NMR (500 MHz, δ, ppm, DMSO-*d*_6_): δ = 10.67 (s, 1H, 1NH, D_2_O exchangeable, Hydrazide), 9.04 (s, 1H, 1NH, D_2_O exchangeable, NHCH_2_CONHNH_2_, amide), 8.99 (1H, aromatic H, C_1_, Pyr.), 8.77 (1H, aromatic H, C_5_, Pyr.), 8.70 (1H, aromatic H, C_3_, Pyr.), 8.50 (s, 1H, 1NH, D_2_O exchangeable, Py–CONH amide), 7.50 (1H, aromatic H, C_4_, Pyr.), 4.24–4.23 (s, 2H, NH_2_), 3.8–3.5 (t, 4H, 2CH_2_, α-gly). MS (EI, 70 eV): *m*/*z* (%) = 252 (M^+^ + 1, 0.59), 251 (M^+^, 7.22), 179 (0.48), 78 (42.94), 63 (100), 49 (2.36). Molecular formula (molecular weight): C_10_H_13_N_5_O_3_ (251.2). Calculated analysis: C, 47.81; H, 5.22; N, 27.87; found: C, 47.80; H, 5.24; N, 27.90.

*The general procedures for the synthesis of Compounds* **5** *and* **6**: A mixture of *N*-(2-(2-hydrazinyl-2-oxoethylamino)-2-oxoethyl)-nicotinamide (**4**) (0.01 mol) and aromatic aldehydes of equimolar amount (namely, *P*-chlorobenzaldehyde and/or *P*-hydroxybenzaldehyde) in ethanol (20 mL) was refluxed for 8 h. The mixture was cooled down and the formed precipitate was filtered off. Finally, the precipitate was recrystallized from dimethylformamide (DMF) to give Compounds **5** and **6**. 

*N-(2-(2-(2-(4-Chlorobenzylidene)-hydrazinyl)-2-oxoethylamino)-2-oxoethyl)-nicotinamide* (**5**): Yield: 60%; melting point: 280–281 °C, IR (cm^−1^): (KBr): ν = 3425 (NH stretching), 2999 (CH, aromatic), 2913 (CH, aliphatic), 1655, 1533 and 1511 (C=O amide I, II, and III, respectively). ^1^H-NMR (500 MHz, δ, ppm, DMSO-*d*_6_): δ = 9.1, 9.09 (s, 2H, 2NH, D_2_O exchangeable, NHCH_2_CONHN=CH–Phe, amide), 8.84 (1H, aromatic H, C_1_, Pyr.), 8.82 (1H, aromatic H, C_5_, Pyr.), 8.72 (1H, aromatic H, C_3_, Pyr.), 8.38 (s, 1H, 1NH, D_2_O exchangeable, Py–CONH amide), 8.36 (d, 1H, N=CH–Phe), 8.29 (1H, aromatic H, C_4_, Pyr.), 7.95–7.15 (4H, the remaining aromatic H), 2.26–2.00 (t, 4H, 2CH_2_, α-gly). MS (EI, 70 eV): *m*/*z* (%) = 373 (M^+^, 10.71), 232 (18.37), 134 (18.03), 77 (33.33), 63 (100), 50 (54.59). Molecular formula (molecular weight): C_17_H_16_ClN_5_ (373.8). Calculated analysis: C, 54.62; H, 4.31; Cl, 9.48; N, 18.74; found: C, 54.64; H, 4.34; Cl, 9.52; N, 18.72.

*N-(2-(2-(2-(4-Hydroxybenzylidene)-hydrazinyl)-2-oxoethylamino)-2-oxoethyl)-nicotinamide* (**6**): Yield: 62%; melting point: 280–281°C, IR (cm^−1^): (KBr): ν = 3420 (OH), 3200 (NH stretching), 2951 (CH, aromatic), 2839 (CH, aliphatic), 1645, 1593, and 1558 (C=O amide I, II, and III, respectively). ^1^H-NMR (500 MHz, δ, ppm, DMSO-*d*_6_): δ = 9.15 (s, 1H, OH, D_2_O exchangeable, aromatic C–OH), 9.12, 9.00 (s, 2H, 2NH, D_2_O exchangeable, NHCH_2_CONHN=CH–Phe, amide), 8.98 (1H, aromatic H, C_1_, Pyr.), 8.62 (1H, aromatic H, C_5_, Pyr.), 8.55 (1H, aromatic H, C_3_, Pyr.), 8.39 (s, 1H, 1NH, D_2_O exchangeable, Py–CONH amide), 8.18–8.16 (d, 1H, N=CH–Phe), 8.08 (1H, aromatic H, C_4_, Pyr.), 7.55–7.40 (4H, the remaining aromatic H), 2.96–2.93 (t, 4H, 2CH_2_, α-gly). ^13^C-NMR (125 MHz, δ, ppm, DMSO-*d*_6_): δ = 173–170 (3CO), 160–122 (11aromatic C), 140.5 (N=CH, Hydrazide), 41.5–40.0 (aliphatic C, 2CH_2_, α-gly). MS (EI, 70 eV): *m*/*z* (%) = 356 (M^+^ + 1, 7.55), 355 (M^+^, 11.05), 224 (6.70), 155 (6.15), 68 (11.14), 56 (100), 50 (14.49). Molecular formula (molecular weight): C_17_H_17_N_5_O_4_ (355.3). Calculated analysis: C, 57.46; H, 4.82; N, 19.71; found: C, 57.45; H, 4.84; N, 19.75.

*The general procedure for the synthesis of Compounds* **7** *and* **8**: A mixture of Compound **4** (0.01 mol), phenyl isocyanate, and/or methyl isothiocyanate (0.01 mol) and triethyl amine as a catalytic amount in dry benzene (20 mL) was refluxed for 7 h. The mixture was thereafter concentrated. The precipitated was filtered off and recrystallized from ethanol to give the new Compounds **7** and **8**.

*N-(2-(2-(2-(Methylcarbamothioyl)-hydrazinyl)-2-oxoethylamino)-2-oxoethyl)-nicotinamide* (**7**): Yield: 77%; melting point: 202–204 °C, IR (cm^−1^): (KBr): ν = 3425 (NH stretching), 3251 (CH, aromatic), 3143 (CH, aliphatic), 1590, 1542, and 1515 (C=O amide I, II, and III, respectively). ^1^H-NMR (500 MHz, δ, ppm, DMSO-*d*_6_): δ = 9.50–8.90 (5s, 5H, 5NH, D_2_O exchangeable, amide), 8.75 (1H, aromatic H, C_1_, Pyr.), 8.60 (1H, aromatic H, C_5_, Pyr.), 8.45, 7.80 (2H, aromatic H, C_3_ and C_4_, Pyr.), 3.50–3.42 (t, 4H, 2CH_2_, α-gly), 2.00 (s, 3H, CH_3_,). ^13^C-NMR (125MHz, δ, ppm, DMSO-*d*_6_): δ = 183 (CS), 165 (3CO), 145–140 (5 aromatic C), 45.7 (aliphatic C, 2CH_2_, α-gly), 31.1 (aliphatic C, CH_3_). MS (EI, 70 eV): *m*/*z* (%) = 325 (M^+^ + 1, 57.45), 324 (M^+^, 63.30), 299 (60.64), 199 (100), 144 (47.87), 75 (57.45), 65 (81.91), 51 (46.28). Molecular formula (molecular weight): C_12_H_16_N_6_O_3_S (324.4). Calculated analysis: C, 44.43; H, 4.97; N, 25.91; S, 9.89; found: C, 44.44; H, 5.00; N, 25.95; S, 9.86.

*N-(2-oxo-2-(2-oxo-2-(2-(Phenylcarbamoyl)-hydrazinyl)-ethylamino)-ethyl)-nicotinamide* (**8**): Yield: 55%; melting point: 230–231 °C, IR (cm^−1^): (KBr): ν = 3290 (NH stretching), 3056 (CH, aromatic), 2677 (CH, aliphatic), 1688, 1660, 1630 and 1599 (C=O amide I, II, III, and IV, respectively). ^1^H-NMR (500 MHz, δ, ppm, DMSO-*d*_6_): δ = 9.22–8.85 (5s, 5H, 5NH, D_2_O exchangeable, amide), 8.80 (1H, aromatic H, C_1_, Pyr.), 8.51 (1H, aromatic H, C_5_, Pyr.), 8.39 (1H, aromatic H, C_3_, Pyr.), 7.62–6.93 (6H, the remaining aromatic H), 3.83–3.66 (t, 4H, 2CH_2_, α-gly). MS (EI, 70 eV): *m*/*z* (%) = 371 (M^+^ + 1, 1.77), 370 (M^+^, 2.79), 322 (100), 236 (4.24), 91 (12.08), 77 (17.96), 64 (43.17), 50 (10.19). Molecular formula (molecular weight): C_17_H_18_N_6_O_4_ (370.4). Calculated analysis: C, 55.13; H, 4.90; N, 22.69; found: C, 55.15; H, 4.94; N, 22.73.

*The general procedure for the synthesis of Compounds* **9** *and* **10**: A mixture of Compound **7** or **8** (0.01 mol) in ethanol (30 mL) and chloroacetic acid (0.01 mol) was refluxed for 10 h. The reaction mixture was concentrated. The precipitate was filtered off and recrystallized from the ethanol to give the corresponding candidates (**9**,**10**), respectively.

*N-(2-(2-(2-(Methylimino)-4-oxothiazolidin-3-ylamino)-2-oxoethylamino)-2-oxoethylamino)-2-oxoethyl)nicotinamide* (**9**): Yield: 70%; melting point: 151–153 °C, IR (cm^−1^): (KBr): ν = 3429 (NH stretching), 2981 (CH, aromatic), 2943 (CH, aliphatic), 1616, 1603 and 1524 (C=O amide I, II and III, respectively). ^1^H-NMR (500 MHz, δ, ppm, DMSO-*d*_6_): δ = 9.40–9.21 (3s, 3H, 3NH, D_2_O exchangeable, amide), 8.72–8.24 (m, 4H, aromatic H, Pyr.), 4.45 (t, 2H, CH_2_, oxazolidine ring), 3.17, 2.95 (t, 4H, 2CH_2_, α-gly), 1.34–1.10 (s, 3H, CH_3_). MS (EI, 70 eV): *m*/*z* (%) = 365 (M^+^ + 1, 2.37), 364 (M^+^, 6.11), 258 (24.53), 73 (100), 57 (25.80), 50 (2.08). Molecular formula (molecular weight): C_14_H_16_N_6_O_4_S (364.4). Calculated analysis: C, 46.15; H, 4.43; N, 23.06; S, 8.80; found: C, 46.17; H, 4.45; N, 23.03; S, 8.85.

*N-(2-oxo-2-(2-oxo-2-(4-oxo-2-(Phenylimino)-oxazolidin-3-ylamino)-ethylamino)-ethyl)-nicotinamide* (**10**): Yield: 80%; melting point: 98–100 °C, IR (cm^−1^): (KBr): ν = 3431 (NH stretching), 3295 (CH, aromatic), 3219 (CH, aliphatic), 1687, 1669, and 1600 (C=O amide I, II and III, respectively). ^1^H-NMR (500 MHz, δ, ppm, DMSO-*d*_6_): δ = 9.12–8.92 (3s, 3H, 3NH, D_2_O exchangeable, amide), 8.28–8.04 (m, 4H, aromatic H, Pyr.), 7.62–6.65 (m, 5H, the remaining aromatic H), 4.29 (t, 2H, CH_2_, oxazolidine ring), 3.67, 3.05 (t, 4H, 2CH_2_, α-gly). ^13^C-NMR (125 MHz, δ, ppm, DMSO-d_6_): δ = 169.2–165.3 (4CO), 156.5 (CH, Oxazolidine ring), 140–118 (11aromatic C), 55.5 (CH_2_, Oxazolidine ring), 40–39 (aliphatic C, 2CH_2_, α-gly). MS (EI, 70 eV): *m*/*z* (%) = 410 (M^+^, 0.26), 322 (100), 235 (0.44), 91 (1.76), 64 (7.69), 50 (1.46). Molecular formula (molecular weight): C_19_H_18_N_6_O_5_ (410.4). Calculated analysis: C, 55.61; H, 4.42; N, 20.48; found: C, 55.64; H, 4.45; N, 20.47.

### 3.2. Biological Evaluations 

#### 3.2.1. Antibacterial and Antifungal Activity (Agar Well Diffusion Assay)

All samples were dissolved in dimethyl sulfoxide (DMSO) at a 1 mg/1 mL concentration in comparison to different antibiotic drugs as standard samples. The strain used and test organisms: A-Bacteria such as *E. coli* (ATCC 25922) and *B. subtilis* (NRRL-B-4219); B-Test fungi such as *A. niger* (ATCC 16888) and *C. albicans* (ATCC 10231). The antimicrobial activity of the candidates (**4**–**10**) was evaluated by use of an agar well diffusion method [40,41]. The samples were dissolved in DMSO. Briefly, a one-day-old culture of bacteria and a two-day-old culture of fungi were then mixed with sterile physiological saline (NaCl 0.9%), and the turbidity was adjusted to the standard inoculums of a MacFarland scale of 0.5 (10^6^ colony forming units/mL). Briefly, agar plates containing 20 mL of Mueller Hinton Agar (Lab M., Bury, Lancashire, UK) and Sabouraud-dextrose agar (Lab M., Bury, Lancashire, UK) were inoculated with bacterial and fungal strains under aseptic conditions and wells (diameter = 9 mm) were filled with 100 µL of the test samples. Standard antibacterial antibiotics, NA = Negram (nalidixic acid), S = Streptomycin; T = Oxytetracycline, VA = Vancomycin, CDZ = Cefodizime, and NV = Novobiocin, and fungal antibiotics, Ny = Nystatin; CLT = Clotrimazole, and FLC = Fluconazole were used as positive controls over bacteria and fungi. A zone of inhibition was recorded after incubating bacterial strains at 37 °C (24 h) and fungal strains at 25 °C, 72 h [42,43].

#### 3.2.2. Determination of the Minimum Inhibitory Concentration (MIC)

The synthesized compounds showed antibacterial activities and were subjected to MIC (minimum inhibitory concentration) assays [44]. Serial dilutions were prepared with concentrations ranging from 100 to 500 μg/mL. DMSO was used as a negative control (Blank DMSO). Each prepared concentration in tubes was applied in plates that were inoculated with 100 μL each of the 10^6^ cfu/mL bacterial and spore suspension from fungal strains, and the assay was applied by an agar well diffusion method. The plates were incubated aerobically at 37 °C (18–24 h) for bacterial strains and 25 °C (48 h) for fungal strains. The MIC values which represent the lowest compound concentration that completely inhibits the growth of microorganisms. All tests were performed in triplicates.

### 3.3. Molecular Docking Studies

The crystal structures of *E. coli* enoyl reducates including triclosan (PDB 1C14) and cytochrome P450 14-𝛼-sterol demethylase (Cyp51) from *Candida albicans* (PDB 5TZ1) were provided from the Brookhaven protein data bank (PDB; http://www.rcsb.org/pdb) and loaded to the Molegro Virtual Docker (MVD 2013.6.0.0 (win32), (Molegro ApS, Aarhus C, Denmark) program. We used a fully functional free trial version with a limited-term license [45]. The non-bonded oxygen atoms of water present in the crystal structure were removed. ChemBio3D Ultra 10 (Billerica, MA, USA) [46] was used to draw the 3D structures of different ligands. The ligands were further pre-optimized using the free version of Marvinsketch 4.1.13 from Chemaxon Ltd. (Budapest, Hungary) [47] with an MM force field and saved in the Tripos mol2 file format. The MolDock score functions were used with a 0.3° grid resolution. The binding sites were defined to any residues that were a distance of 12 Å away from the co-crystallized tetrazole-based antifungal drug and fluconazole in the complex crystal structures of the enzymes [48,49,50,51].

## 4. Conclusions

In the present study, seven novel dipeptide Compounds **4**–**10** were synthesized using a solution phase method in peptide synthesis. The compounds were evaluated for their antibacterial activity against Gram-positive, Gram-negative, and fungi by the agar well diffusion method, all the synthesized candidates showed a significant antimicrobial inhibitor activity. Docking studies have been carried out on enoyl reeducates from *E. coli* and P450 14-α-sterol demethylase (Cyp51) from *Candida albicans* active sites. The MolDock scores of the seven tested compounds range between −117 and −171 and between −179 and −107, respectively. 

## Figures and Tables

**Table 1:** The antimicrobial activities of the synthesized candidates expressed as inhibition zones of growth in millimeters against the used test organisms.

Compounds	Test Organisms			
	Bacteria		Fungi	
	Gram-Positive	Gram-Negative	Unicellular	Filamentous
	*B. subtilis*	*E. Coli*	*C. albicans*	*A. niger*
**Inhibition zone (mm)**				
**4**	29	30	28	16
**5**	20	19	25	20
**6**	16	15	17	00
**7**	20	20	18	00
**8**	18	17	18	00
**9**	30	15	30	00
**10**	20	20	18	00
**Standard Antimicrobial Antibiotics**				
NA = 30 µg	20	16	00	00
S = 10 µg	14	00	12	00
NV = 30 µg	29	30	00	00
T = 30 µg	30	27	00	00
CDZ = 30 µg	00	20	00	00
VA = 30 µg	21	23	00	00
Ny = 100 µg	00	00	14	15
CLT = 50 µg	00	00	12	10
FLC = 25 µg	00	00	13	11

Standard bacterial and fungal antibiotics. NA = Negram (nalidixic acid); S = Streptomycin; NV = Novobiocin; T = Oxytetracycline; CDZ = Cefodizime; VA = Vancomycin; Ny = Nystatin; CLT = Clotrimazole; FLC = Fluconazole.

**Table 2:** MolDock scores of the tested compounds.

Compounds	Moldock Score with Enoyl Reductase from *E. coli* (PDB 1C14)	Moldock Score with Cytochrome P450 14-α-Sterol Demethylase (Cyp51) from *Candida* (PDB 5TZ1)
**4**	−117	107
**5**	−147	147
**6**	−156	151
**7**	−139	140
**8**	−153	161
**9**	−142	151
**10**	−171	179
Reference	−127 (triclosan)	133 (fluconazole)
